# Diabetic retinopathy service delivery and integration into the health system in Pakistan—Findings from a multicentre qualitative study

**DOI:** 10.1371/journal.pone.0260936

**Published:** 2021-12-15

**Authors:** Stevens Bechange, Anne Roca, Elena Schmidt, Munazza Gillani, Leena Ahmed, Robina Iqbal, Imran Nazir, Anna Ruddock, Muhammed Bilal, Itfaq Khaliq Khan, Sandeep Buttan, Emma Jolley

**Affiliations:** 1 Sightsavers Pakistan Country Office, Islamabad, Pakistan; 2 Department of Policy and Programme Strategy, Sightsavers, Haywards Heath, United Kingdom; 3 Sightsavers India Country Office, New Delhi, India; International Medical University, MALAYSIA

## Abstract

This paper is based on qualitative research carried out in a diabetic retinopathy (DR) programme in three districts of Pakistan. It analyses the organisation and delivery of DR services and the extent to which the interventions resulted in a fully functioning integrated approach to DR care and treatment. Between January and April 2019, we conducted 14 focus group discussions and 37 in-depth interviews with 144 purposively selected participants: patients, lady health workers (LHWs) and health professionals. Findings suggest that integration of services was helpful in the prevention and management of DR. Through the efforts of LHWs and general practitioners, diabetic patients in the community became aware of the eye health issues related to uncontrolled diabetes. However, a number of systemic pressure points in the continuum of care seem to have limited the impact of the integration. Some components of the intervention, such as a patient tracking system and reinforced interdepartmental links, show great promise and need to be sustained. The results of this study point to the need for action to ensure inclusion of DR on the list of local health departments’ priority conditions, greater provision of closer-to-community services, such as mobile clinics. Future interventions will need to consider the complexity of adding diabetic retinopathy to an already heavy workload for the LHWs.

## Introduction

Globally, an estimated 463 million people live with diabetes mellitus (DM). More than 70% of these are in low and middle-income countries (LMICs) [1]. Diabetic retinopathy (DR) is a common ocular complication of diabetes that requires an early diagnosis, routine monitoring and treatment to prevent vision loss and blindness. The Global Diabetic Retinopathy Advocacy Initiative group promotes the integration of DR management into the health system as a regular diabetes service [2]. The Initiative recommends DR screening for all diabetic patients who make contact with the system. However, due to various factors affecting both demand and supply [3], eye care for many patients remains suboptimal and many develop severe and irreversible vision impairment. The existing literature on the implementation of integrated DM/DR service models in LMICs is very limited. There is scant evidence on the experiences of patients and healthcare providers involved in these models and there are knowledge gaps on how to make DM/DR services more patient centred.

The Pakistan National Diabetes Survey carried out in 2016–17 estimated the prevalence of diabetes among adults aged 20 years and above at 26.3% [4]. Only 19% of these people were aware of their diagnosis at the time of the survey [4]. A systematic review published in 2018 estimated the pooled prevalence of DR among people with diabetes in Pakistan at 28.8% and vision threatening DR (VTDR) at 28.2% of all DR and 8.6% of all people with diabetes [5]. However, the majority of the studies in the review were done in hospital settings with only four studies conducted in the community. Prevalence of DR in the community- based studies was low but the authors cautioned that it was likely to be an underestimate because community-based studies record only the primary cause of vision impairment (VI), which in many cases is cataract present alongside DR [6, 7]. Therefore, the true burden of DR in Pakistan is likely to be much higher than reported and all DM patients are recommended to attend DR screening to identify retinopathy as early as possible.

The study presented here was conducted alongside a project delivered by Sightsavers, an international non-governmental organisation and its partners. The project was designed to link DR screening and treatment with existing diabetes clinics in three tertiary hospitals in Lahore and Rawalpindi, Punjab province and in Karachi, Sindh province. The project included interventions at three levels. In the community, Lady Health Workers (LHWs) and general practitioners were trained to raise community awareness and encourage patients to take up DM and DR screening. Similar type of training was also delivered for GPs and medical technicians in primary care centres (Basic Health Units and Rural Health Centres). At the tertiary level, optometrists and DM counsellors were trained on DM/DR referrals and all patients with confirmed diabetes were referred and screened for DR. Patients with DR and sight threatening DR were referred to an ophthalmologist. Those who did not have DR were advised to come back for follow up checks annually or bi-annually. Ophthalmologists underwent specialist surgical skills training. Supplies and equipment for screening, referral and DR management were provided throughout the lifetime of the project. The project also supported the establishment of a patient tracking system, patient counselling services and facilitated linkages between different hospital departments.

This study aimed to explore the implementation approaches adapted by the project facilities and the experiences of patients and healthcare providers in receiving and providing the DM/DR care. This paper focuses specifically on i) the role of Lady Health Workers in DM/DR care; ii) referral pathways and uptake; iii) the organisation of DM/DR services in three tertiary hospitals involved in the project; and iv) challenges in accessing DM/DR care experienced by patients.

## Methods

### Study settings

The three hospitals participating in the study were located in urban areas but were different in size, capacity and ownership. In Karachi, the project was based at Al-Ibrahim Trust Eye Hospital (AIEH), a specialist private not-for-profit health facility, where all DR services from screening to treatment are provided within the same premises. The diagnostic laboratory and pharmacy services are also available on site. Patients requiring additional services such as gynaecology or cardiology, are referred to another hospital located in an adjacent building. In Lahore, the project worked with Mayo hospital, a leading public sector hospital in Punjab province, which is also a teaching hospital for the King Edward Medical College (KEMC)—a well-regarded medical university in Pakistan. All professors and senior surgeons of the College work at the hospital. This is a very large hospital with many departments and facilities. Screening for DR is conducted at the outpatient department (OPD). Patients diagnosed with DR are referred for management to the eye department located about one kilometre away from the OPD. In Rawalpindi, the project was based at the Holy Family Hospital (HFH), one of the three public sector hospitals in the district. It is located in the centre of the city and provides a wide range of health services to patients from Rawalpindi and neighbouring towns. DM and DR services are available in the same building, on the ground floor with accessible pathways.

### Study design

The study had a qualitative design utilising focus group discussions (FGDs) and in-depth interviews (IDIs), as data collection methods. FGDs were conducted with patients and Lady Health Workers. IDIs were carried out with health professionals at both primary and tertiary facilities, including project staff, patients, LHWs and their supervisors. FGDs generated a range of themes, which were further explored during IDIs. The study was conducted between January and April 2019.

### Sampling and participant recruitment

To capture a range of experiences, the study sought to recruit a diverse group of purposively selected participants: women and men, people of different ages, patients with different stages of DM and DR, health workers, managers and civil society stakeholders. Data from the hospital databases were stratified to identify patients with different characteristics and different treatment adherence patterns.

### Data collection, management, and analysis

The topic guides (S1 Table) centred around participants’ views on the DM/DR project, organisation of services, patient referral and treatment pathways, health provider roles, attitudes and patient-provider relationships.

We conducted 14 FGDs and 37 IDIs with 144 participants. In-depth interviews and FGDs took place during the first quarter of 2019 and were completed by two interviewers (one female and one male) in Urdu or Punjabi. Interviews took place at the participating hospitals or patients’ homes and, were face to face. They lasted between 30 and 90 minutes. Prior to each interview or group discussion, the researchers introduced themselves and explained the study. The interviewers were trained MSc-level social scientists who were not involved in the project. Most IDIs and FGDs were audio-recorded, however, two participants were not willing to be recorded, so notes were taken during these interviews.

All audio recordings were transcribed and translated into English. Data were analysed using NVIVO software version 12 [8]. The coding of the data was undertaken by the two interviewers and two study co-investigators. After reading the first batch of transcripts independently, the team collaboratively developed a codebook, which was tested with the next batch of transcripts, discussed and adjusted. All the remaining transcripts were then coded by two members of the team using the final version of the codebook. The compare and contrast approach was used to identify themes and sub-themes [9]. Interactive discussions were held within the team to validate data interpretation and resolve any discrepancies. A list of agreed themes and sub-themes was finalised in the light of the research objectives.

### Ethical considerations

The study protocol was reviewed and approved by the research ethics committees in the three districts: the Institutional Review Board (IRB) of Isra Postgraduate Institute of Ophthalmology Karachi (protocol #: A-00088), the IRB of the College of Ophthalmology and Allied Visual Sciences at Mayo Hospital, Lahore (ref #: COAVS/73/19) and the IRB of the Rawalpindi Medical College (ref #: R.73/RMU). All participants were informed about the purpose of the study, had an opportunity to ask questions and provided an informed written consent.

## Results

### Participant characteristics

A total of 144 participants (Rawalpindi = 43, Lahore = 45, Karachi = 56) were interviewed in this study, including 98 patients, 20 LHWs and 26 clinicians, managers and other stakeholders. Among the 98 patients, 41 were female and 57 were male. Their ages ranged from 40 to 73 years with a median age of 59 years. Five patients were single, and the rest were married. Most patients had been aware of their diabetes status for two to eight years, with the longest period being 20 years. Nearly three-quarters of the patients interviewed did not work, mainly due to diabetes related health issues. A few were engaged in small-scale businesses and trades and only four patients were in formal salaried employment.

All twenty LHWs interviewed reported a minimum of 10 years of formal education. Their median age was 38 years ranging from 26 to 56 years. LHWs from Karachi were younger than those from Lahore or Rawalpindi. Most had been working as LHWs for three to 10 years; a few had been in their role for more than 15 years.

Among other participants, ten were eye care specialists and nine were clinicians from other hospital departments involved in diabetes care, including four in senior leadership roles at their hospitals. Other stakeholders included two programme counsellors; two project managers and three behavioural/social science specialists involved in the project.

The findings that emerged from the interviews are organised in four overarching themes as summarised in Table 1 below.

**Table 1:** Overview of themes and subthemes.

Theme	Sub-themes and illustrative examples	Some illustrative quotes
**Role of LHWs in diabetic care**	***Encouraging patients to seek care early on and adhere to treatment***	*LHWs are best placed to provide awareness and information so that patients don’t report to us when the disease has got complicated*. [Consultant Surgeon]
	Limited interactions with men, male patients did not clearly understand the role of LHWs in diabetic care	

	LHWs felt that the uptake of services could be improved if they could find a way to engage more men, and other influential people in the community.	*We haven’t talked to them [men]*. *We need to find a way*. *The man’s wife tells us about them*, *for example they would ask us to give them some medicine for their husband who has sugar [diabetes]*… [Lady Health Worker]
	Training received generally increased LHWs knowledge and skills in guiding and counselling patients.	
	Lack of understanding of what happens when patients visit the hospitals.	

	In some cases, LHWs told patients that a referral slip by a LHW would allow them to skip the queue at the hospital. This information was incorrect, which frustrated patients who had to spend hours queuing at the hospital and in some cases, returned home without seeing a doctor.	*I had this man and another woman who visited the hospital*. *I sent them and [they] came back to the community at night without seeing a doctor*. *They said to me and other people in the community that our slip had no value*, *that it was a waste of time and money to go there and line up for the whole day*, *and no doctor sees you*. [Lady Health Worker]
	Some patients felt that the LHWs in their community did not dedicate sufficient time with patients to explain the problem of diabetes and related risks.	
	LHWs felt that there was little attention given to eye health more broadly, and highlighted a lack of visible eye health promotion campaigns in the media, in contrast to the campaigns organised for polio, dengue fever or reproductive health.	
	***Collection of basic health data***	
	LHWs activities focus on the issues identified by the government as health priorities and those that are required in their monthly reports	*We focus on those priority activities that the government requires us to monitor and report*. *Eyes are not in the health department priority list*… [Lady Health Supervisor]

	There was no government requirement to monitor and report the DR referral uptake.	
	Role of LHWs in eye care could be improved if eye health was included in the Department of Health priority list and if LHWs were required to report eye health indicators as part of their routine activities.	

	***Provision of basic health services***	
	Some LHWs felt that there was confusion about what services they were expected to provide in diabetes and eye care. Some patients for example thought LHWs should have the necessary equipment for measuring blood pressure and glucose level and carry the equipment to home visits in the same way they carry scales to weigh babies.	*They [LHWs] don’t come with the machine to measure my blood sugar*. *I have requested them to come with it*, *but they always only come with the other machines for weighing the babies*. [Female Patient]
	LHWs also felt overburdened with a range of duties and responsibilities and even though many LHWs were in principle interested to provide more eye related services, there were concerns about feasibility of doing it effectively within the time and resources available	
		*It is a lot of work for us*. *Polio*, *other vaccinations*, *mobilisations*. *Everything which comes the government puts on us*, *but we are also wives and mothers with other work at home*. [Lady Health Worker]
	Other healthcare stakeholders also expressed concerns about LHWs’ workload and the limited support systems available to them.	
**Referral pathways and uptake**	***LHWs at the community level referred suspected diabetic cases to DM/DR services***.	

	Very few patients referred by LHWs took up their referral straight away.	
	Many referred patients, both men and women, tended to visit another healthcare practitioner at the community or district level for ‘a more professional opinion’ before making a trip to the DM/DR facility.	*She mentioned that I have diabetes*, *that I should go to hospital for eye check-up*. *She just noted the information*, *told me a little bit about it and left*. *But going to hospital just like that is time consuming and there is cost*, *so before I came here*, *I had to consult another doctor in the community who knows more*. [Male Patient]
	***GPs*, *MOs and MTs working at the community and primary care facilities were also trained to identify and refer patients***	
	Majority of patients referred by GPs, MOs or MTs took up their referrals.	*I went for a check-up at the free outreach camp*. *Over there*, *the doctor checked my eyes and personally told me to go to the hospital for treatment before things get worse*. *So*, *I knew it was serious*, *as the doctor had explained well and today*, *here I am [at the hospital]*. [Female Patient]
	Patients pointed out that they trusted the opinion of the medical personnel and treated their referral as a matter of urgency.	
	Some patients noted further that the health care personnel who they consulted explained potential health complications of diabetes well and answered the questions the patients asked.	
		*They talk to us in a polite manner and the doctors are very cooperative*. *Especially*, *the madam*. *She is very understanding and cooperative*. *The check-up is very professional and the prescription of medicines too*. *…*. *the tests are being done at 4000 rupees elsewhere but here it is just 700 rupees*. [Male Patient]
	***Walk-in patients***	
	Brought in the highest number of DM/DR cases.	
	A large number of patients (>50 per day), many of whom were walk-ins, attended the hospital based on recommendations of other patients (Karachi).*	
	Patients described their positive experiences with hospital and good quality services delivered at subsidised prices making eye care affordable for poorer patients (Karachi).	*The truth is that when I came here on Friday*, *they took very good care of me*. *The main point is that my time and money was not wasted here*. [Female Patient]
**Organisation of services**	***Private not-for-profit hospital in Karachi***	

	Positive perception of health facility and services.	
	Time- and cost-efficient with different types of examinations and tests carried out in a short span of time and the laboratory results often available on the same day.	
	***Public sector hospitals in Lahore and Rawalpindi***.	*As a diabetic person*, *I need to use the washroom often but can get to it easily here*. *there so many patients here*, *and the corridors are very narrow*. [Male Patient]
	Often overcrowded with more than 100 patients attending every day.	
	Narrow corridors, making the movement of elderly patients and wheelchair users challenging (Lahore).	

	Patient frustration with having to walk long distances between units located more than a kilometre apart.	
	***Utility of the patient tracking systems***	
	The system was linked with the hospital databases and used to generate lists of patients scheduled for follow up two days before their appointment.	*This [follow up calls] is a good thing*, *and in case the husbands are strict and don’t let their wives out*, *then one can call the husband’s number repeatedly and send them text messages*. *Only then*, *the husbands get worried that the doctor is calling them and there has to be a serious case for it*. *Otherwise*, *they think that their wives do it intentionally just to wander about in the hospital or somewhere else or to avoid work*. [Female Patient].
	Patients reported that the messages or phone call reminders were very helpful to ensure the visit to the hospital on the scheduled date.	
	***The role of counsellors***	
	All patients who came to these facilities for DR/DM services saw a counsellor.	
	Patients reported a high degree of appreciation for the role of the counsellors as their main contact in seeking advice on the day-to-day management of diabetes and eye related problems.	*If she [counsellor] explains something and you don’t understand it*, *you can just ask her again and she even explains the same thing three times to make you understand*. [Male Patient]
	***Availability and cost of medicines at the project facilities***	
	Patients reported that when prescribed medicines at the hospital, they were asked to procure them from private vendors outside the hospital, at high and often variable prices.	*… the problem is that all the medicines are distributed as we have high turnout of patients and in that case*, *they [patients] have to buy them from the market*. [Doctor]
	Medicines for diabetes and eye diseases were available to hospital patients at discounted prices, as were glucometers and diabetic strips to test blood sugar level (Karachi)	

**Challenges of DM/DR care reported by patients**	***Financial constraints***	
	The majority of patients interviewed were unemployed.	
	For many, basic household needs such as housing, food and children’s education, were of higher priority than healthcare.	
	Many people could not afford medical consultations and reported buying medicines from pharmacies on an ad-hoc basis or using homemade and herbal remedies.	
	hospitals tried to be sensitive and responsive to the patients’ financial circumstances and made arrangements for financial support (Karachi).	*Only last day [yesterday] they told … me 6500 rupees [approximately US$ 40] will be charged*. *But I pleaded to them that I am a poor man*. *Then they gave me a slip*, *it was filled with the reduced fee*, *and I deposited 4000 rupees [US$ 25]*. [Male Patient]
	***Busy hospitals and long waiting times***	
	Long queues and waiting times (>6 h) affecting livelihood demands.	
	Waiting long hours in a queue was painful for patients with advanced diabetes.	*But when we have to spend 6 to 7 hours waiting and don’t get treatment*, *and told to come back the next day then it is useless*. [Male Patient]
	Patients, who needed only simple routine monitoring said that they preferred visiting community GPs, who were available in the evenings and could check blood pressure and sugar level at a small fee.	
	***Difficulties with travel and transportation***	
	Availability of public transportation was reported to be a major factor in the uptake of referrals.	
	The costs of private taxis was reported to be too high, and the majority of patients could not afford them.	
	Female patients were reported to have additional barriers with travelling, as they were required to get permission from their husbands to visit the hospital and also needed someone to accompany them.	*In our community*, *females cannot move alone*. *I do not want to come with anyone other than my son or daughter in law*. *This was the reason for the delay*. [Female Patient]

	***Lack of awareness about eye related complications***	
	Even those who were reasonably well educated, did not seem to be aware of eye related complications of diabetes.	*I did not know*. *It is the lady health worker who told me that the problem I had in my eyes was because of sugar*, *my sugar was not getting under controlled*. *So*, *she asked me to come here*. *They checked my eyes yesterday and have suggested laser for both eyes*. [Female Patient]

LHW: lady health worker; GP: general practitioner; MO: medical officer; MT: medical technician; DM: diabetes mellitus; DR: diabetic retinopathy. *Where an issue was mentioned in one study site, this is marked with the district name.

### The role of lady health workers in diabetic care

One of the objectives of the project was to increase the role of Lady Health Workers in diabetic care by improving the ways through which they identified suspected diabetic cases and encourage them to go for screening in tertiary health facilities. LHWs play a critical role in the delivery of primary and community healthcare in Pakistan, by collecting basic health data, providing some basic interventions and raising awareness of health services and disease control programmes. The relationship is particularly strong with women, as a large number of services provided by LHWs relate to maternal and child health (MCH). Many female patients interviewed in this study pointed out to the high degree of confidence and trust they had in LHWs and often referred to them using the name ‘Baji’, which means ‘elder sister’.

The arrangement with the DR project was that LHWs would receive basic training on DM and DR and refer known or suspected diabetic patients to the project hospitals or other health facilities with DM/DR screening services. The eye care professionals interviewed in the study were confident that if appropriately trained, LHWs could play an important role in early identification of DM and prevention of DM complications, including DR. The critical aspect of their role from the eye care professionals’ perspective, was encouraging patients to seek care early on and adhere to treatment.

“*LHWs are best placed to provide awareness and information so that patients don’t report to us when the disease has got complicated*”.

***[Consultant Surgeon, Karachi]***

LHWs interviewed said that the training by the project was useful. It increased their knowledge on the importance of monitoring blood sugar levels as well as on eye related complications of diabetes. The training also increased LHWs’ skills in guiding and counselling patients. However, many LHWs highlighted a considerable gap in their knowledge regarding the work of the DM/DR units. Many did not know what would happen during the visits to the tertiary hospitals and could not answer patients’ questions about the screening. In some cases, patients were told that a referral slip by a LHW would allow them to skip the queue at the hospital. This information was incorrect, which frustrated patients who had to spend hours queuing at the hospital and in some cases, returned home without seeing a doctor. One LHW explained the situation faced by two of her referred patients:

“*I had this man and another woman who visited the hospital*. *I sent them and [they] came back to the community at night without seeing a doctor*. *They said to me and other people in the community that our slip had no value*, *that it was a waste of time and money to go there and line up for the whole day*, *and no doctor sees you*”

***[Lady Health Worker, Karachi]***

Among patients interviewed, many did not think that the LHWs trained by the project had good knowledge of diabetes or were prepared to advise patients on DR screening and treatment. In addition, the LHWs participating in the study had limited interactions with men and many male patients did not clearly understand the role of LHWs in diabetic care. Men knew about LHW activities only through their wives or other women in the family and almost all male patients involved in the study were referred to the DM/ DR facilities by GPs or medical officers or were walk-ins, as one male patient explained:

“*LHWs do visit for administering polio drops*, *but they have never enquired about any other things…*. *No*, *not a single time*. *They have not checked our sugar… They only visit our homes to administer polio vaccine and ask … how many people live here*.”

***[Male patient, 68 years, Rawalpindi]***

Male participants stressed that it was very important for LHWs to engage with men, particularly male elders, to raise awareness of diabetes complications, as many people in the community listened to the elders. LHWs themselves also said that the uptake of screening services could be improved if they could find a way to engage more men, religious scholars, councillors and other influential people in the community.

“*We haven’t talked to them [men]*. *We need to find a way*. *The man’s wife tells us about them*, *for example they would ask us to give them some medicine for their husband who has sugar [diabetes]*. *We tell them that they should take their husband to a good doctor for his sugar and his eye check-up*, *if he has any eye problems*”.

***[Lady Health Worker, Karachi]***

Some participants also said that the LHWs in their community did not dedicate sufficient time with patients, including female patients, to explain the problem of diabetes and related risks. They wanted LHWs to take into account that some patients were old and uneducated, which required more time and effort to encourage them to go to hospital:

“*They [LHWs] don’t do their duty like it should be done*. *There are women that are old*, *ill*, *and not educated*. *LHWs should tell [them] about health practices*, *and I think this is the duty of LHWs… but they don’t do their work the way it is supposed to be done*.”

***[Male patient, 50 years, Rawalpindi]***

LHWs highlighted a number of challenges in the delivery of diabetes messages and DR referrals. First, many LHWs said that their activities focused on the issues identified by the government as health priorities and those that were required in their monthly reports. However, there was no government requirement to monitor and report the DR referral uptake. LHWs felt that there was little attention given to eye health more broadly, and highlighted a lack of visible eye health promotion campaigns in the media, in contrast to the campaigns organised for polio, dengue fever or reproductive health. Several respondents said that the role of LHWs in eye care could be improved only if eye health was included in the Department of Health priority list and if LHWs were required to report eye health indicators as part of their routine activities.

LHWs also mentioned that they felt overburdened with a range of duties and responsibilities and even though many LHWs were in principle interested to provide more eye related services, there were concerns about feasibility of doing it effectively within the time and resources available. Other healthcare stakeholders also expressed concerns about LHWs’ workload and the limited support systems available to them.

Some LHWs also said that there was confusion about what services they were expected to provide in diabetes and eye care. Some patients for example thought LHWs should have the necessary equipment for measuring blood pressure and glucose level and carry the equipment to home visits in the same way they carry scales to weigh babies.

A number of LHWs mentioned a shortage of referral slips, although some questioned whether these slips were necessary at all, as hospitals did not give much attention to the slips issued by LHWs and patients preferred to present a slip issued by a GP or a medical officer and often visited them to get a ‘proper’ referral slip.

“*Patients say that our slip should have value*, *so that they don’t make them wait there [at the hospital]*. *That doesn’t happen …*, *one has to wait in a line*. *But patients think it is our referral with no value*, *so they sometimes go and get a referral slip from a doctor which they go with to the hospital*”.

***[Lady Health Worker, Karachi]***

### Referral pathways and uptake

Participants described four different pathways through which diabetic patients were identified and referred to DR screening in the models of care supported by the project. First, at the community level, LHWs and general physicians (GPs) referred suspected diabetic cases to DM/DR services for further examination and treatment. Second, GPs, medical officers (MOs) and medical technicians (MTs) working at primary care facilities were also trained to identify and refer patients they came across. The third source of referrals was through endocrinology and general medicine departments or OPD clinics of the public sector tertiary hospitals, including the two hospitals included in this study. The fourth referral route which brought in the highest number of DM/DR cases, particularly to the private eye care hospital in Karachi, was walk-in patients.

Study participants explained that the uptake of referrals to DM/DR screening varied depending on who referred the patient and where he or she was referred to. The majority of patients referred by GPs, MOs or MTs took up their referrals. Patients pointed out that they trusted the opinion of the medical personnel and treated their referral as a matter of urgency. Some patients noted further that the health care personnel who they consulted with explained potential health complications of diabetes well and answered the questions the patients asked.

“*I went for a check-up at the free outreach camp*. *Over there*, *the doctor checked my eyes and personally told me to go to the hospital for treatment before things get worse*. *So*, *I knew it was serious*, *as the doctor had explained well and today*, *here I am [at the hospital]*”.

***[Female patient, 50 years, Rawalpindi]***

In contrast, very few patients referred by LHWs took up their referral straight away. Many referred patients, both men and women, tended to visit another healthcare practitioner at the community or district level for *‘a more professional opinion’* before making a trip to the DM/DR facility. In two instances, patients first consulted a healthcare provider in a nearby public hospital, followed by a consultation in a private hospital before they eventually decided to travel to the DM/DR unit. To explain this behavior, study participants referred to mistrust in guidance given by the LHWs:

“*She mentioned that I have diabetes*, *that I should go to hospital for eye check-up*. *She just noted the information*, *told me a little bit about it and left*. *But going to hospital just like that is time consuming and there is cost*, *so before I came here*, *I had to consult another doctor in the community who knows more*”.

***[Male patient, 60 years, Lahore]***

The number of patients taking up referrals also varied across the three participating hospitals. The private hospital in Karachi reported a large number of patients (>50 per day), many of whom were walk-ins, who attended the hospital based on recommendations of other patients. Most patients interviewed in the study described their positive experiences with this hospital and praised it for good quality services delivered at subsidised prices making eye care affordable for poorer patients.

“*They talk to us in a polite manner and the doctors are very cooperative*. *Especially*, *the madam*. *She is very understanding and cooperative*. *The check-up is very professional and the prescription of medicines too*. *…*. *the tests are being done at 4000 rupees elsewhere but here it is just 700 rupees*”.

***[Male patient, 48 years, Karachi]***

The two public hospitals participating in the study received comparatively lower number (<12 per day) of referrals and walk-in patients. In Rawalpindi, the hospital was located in an area with many other charity eye hospitals, which are known to provide good quality inexpensive healthcare services. The health workers interviewed said that they had more patients from other, more distant locations than those referred from the project catchment areas.

Mayo Hospital in Lahore, despite being a large busy public hospital, also received a low number of DR referrals with some participants suggesting less than 10% of referred patients taking up the referral. The main explanation was also competition with other public and charity hospitals available in the area.

### Organisation of services at the hospitals

#### Private not-for-profit hospital in Karachi

Patients interviewed in the study felt that the organisation of service delivery in this hospital was time- and cost-efficient with different types of examinations and tests carried out in a short span of time and the laboratory results often available on the same day. Beds and food at the facility were reported to be free or subsidised depending on the financial circumstances of individual patients.

“*The truth is that when I came here on Friday*, *they took very good care of me*. *The main point is that my time and money was not wasted here*”.

***[Female patient, 66 years, Karachi]***

#### Public sector hospitals in Lahore and Rawalpindi

Both healthcare workers and patients interviewed pointed out that the OPD facility at the two government hospitals were very busy and often overcrowded with more than 100 patients attending every day. The corridors at the hospital in Lahore, in particular were reported to be too narrow, making the movement of elderly patients and wheelchair users challenging. The seating arrangements were thought to be inadequate to accommodate large numbers of patients.

It was further described that patients with suspected VTDR were referred to the eye department for management; the unit is located in the same compound but about one kilometre away from the OPD. Some patients reported difficulties in walking such a distance and some decided not to go to the eye department, as prescribed. The distance was reported by the health workers to be particularly challenging for patients with severe diabetes, elderly patients and patients with disabilities.

Study participants explained that at both public hospitals, there were separate wards for male and female patients to ensure comfort and privacy. The wards were reported to be reasonably clean and equipped with basic equipment for everyday care. Beds and food were reported to be provided free to all patients admitted in the wards. However, access to washrooms, a basic necessity for diabetic patients, was deemed inadequate, as located far away from the ward, unclean, and usually crowded.

Despite these challenges, many participants reported that they were generally happy with the services they had received, felt that their vision was cared for and that they had fewer eye related problems as a result. Patients also praised the professionalism of healthcare workers at the hospitals. This was not surprising as patients are sometimes reluctant to report poor behaviour by doctors, given social hierarchies. Many patients said that the premises were overcrowded and sometimes dirty, but the doctors were experienced, knowledgeable and skilful. They reported that prior to the surgery, the healthcare staff had carefully monitored their condition. Patients’ sugar level and blood pressure were also monitored. It was reported that the doctors would not carry out any surgical interventions until the patient was physically stable and psychologically ready for the treatment.

“*Before operation*, *they check blood pressure and diabetes… They have carried out strict monitoring before the eye operation”*.

***[Female patient, 70 years, Rawalpindi]***.

#### Utility of the patient tracking systems

At all three facilities, the project set up a patient tracking system. The system was linked with the hospital databases and used to generate lists of patients scheduled for follow up two days before their appointment. The system was reported to be useful in sending reminders to the patient mobile phones.

“*Yes*, *we do have a system for calling back*. *This system generates message reminders when six months or one year follow ups are due… The counsellor receives this … on his email … a day before the due date*. *He calls … to remind them [patients] about the follow up…*”.

***[Healthcare worker, Mayo Hospital, Lahore]***

Patients reported that the messages or phone call reminders were very helpful to ensure the visit to the hospital on the scheduled date. Female patients found them particularly helpful, as they needed to make multiple arrangements before their visit, get permission from their husband, organise childcare and find transport and a companion.

“*This [follow up calls] is a good thing*, *and in case the husbands are strict and don’t let their wives out*, *then one can call the husband’s number repeatedly and send them text messages*. *Only then*, *the husbands get worried that the doctor is calling them and there has to be a serious case for it*. *Otherwise*, *they think that their wives do it intentionally just to wander about in the hospital or somewhere else or to avoid work*.”

***[Female patient, 40 years, Rawalpindi]***.

Patients waiting for surgeries were also reminded to report to the health facility using the tracking system. Ophthalmologists praised this tracking system and said it was critical for prevention of DR and VTDR.

#### The role of counsellors

The project also supported a counselling service at all three project facilities. The reminder calls were the opportunity for the counsellors to engage with the patients and encourage them, if needed, to attend their scheduled appointments. All patients who came to these facilities for DR/DM services saw a counsellor. Patients reported a high degree of appreciation for the role of the counsellors as their main contact in seeking advice on the day-to-day management of diabetes and eye related problems.

“*If she [counsellor] explains something and you don’t understand it*, *you can just ask her again and she even explains the same thing three times to make you understand*”.

***[Male patient, 48 years, Karachi]***

#### Availability and cost of medicines at the project facilities

One of the main reasons many patients visited public sector hospitals was the availability of free medicines at these facilities. However very often, these hospitals experienced stock-outs. Patients reported that when prescribed medicines at the hospital, they were asked to procure them from private vendors outside the hospital, at high and often variable prices.

“*… the problem is that all the medicines are distributed as we have high turnout of patients and in that case*, *they [patients] have to buy them from the market*”.

***[Doctor, Mayo Hospital, Lahore]***

Although free medicines at public hospitals were generally appreciated, some patients raised concerns about the quality of medicines offered at cheaper prices or free of charge.

Surgeries in the public hospitals participating in the project were also provided free of charge. Surgical kits and eye lenses were available for free for those who could not afford to pay. Those patients who could afford buying a surgical kit or lens, were asked to buy them from the private pharmacies, so that the poorest patients could be subsidised and receive a completely free surgery.

The private hospital in Karachi had its own pharmacy within the hospital premises. All types of medicines for diabetes and eye diseases were available to hospital patients at discounted prices, as were glucometers and diabetic strips to test blood sugar level.

#### Inter-departmental linkages and opportunities for integration

The DM/DR project aimed to strengthen linkages with other clinical departments at the participating hospitals as part of an effort to ensure continuity of care and include care for other diabetes complications such as neuropathy and gestational diabetes.

To do so, the DM/DR project team engaged with the heads of other departments. Several participants pointed out that these relationships were helpful for patients who were able to access a comprehensive package of services in one hospital, saving trips to multiple hospitals or private laboratories for various tests. The patient referral system was reported to work both ways: patients from the DM/DR unit were referred to other services, while patients visiting other departments were referred to the DM/DR unit.

“*I am not aware of the system the hospital was following earlier… Now*, *every department is aware*, *they have information*. *They keep referring their patients*. *We keep on sending them biannual reminding letters through our heads*.”

***[Manager, Mayo Hospital, Lahore]***

The project team also sent DM/DR specific information and education materials to other departments to ensure that they had the necessary information and referred their patients for screening.

### Challenges of DM/DR care reported by patients

#### Financial constraints

Income was reported to be one of the major factors affecting individual lifestyles and health seeking behaviour of people in the studied communities. The majority of patients interviewed were unemployed. For many, basic household needs such as housing, food and children’s education, were of higher priority than healthcare. Many people could not afford medical consultations and reported buying medicines from pharmacies on an ad-hoc basis or using homemade and herbal remedies. Most patients, irrespective of their age and sex, reported delaying visits to healthcare professionals until the condition worsened or pain became unbearable, as one female patient explained:

“*We are a poor family*. *My son drives a rickshaw and when he has time and money*, *he brings me [to the hospital]*, *if the pain becomes unbearable*.”

***[Female patient, 60 years, Rawalpindi]***.

However, a number of patients said that the hospitals tried to be sensitive and responsive to the patients’ financial circumstances and made arrangements for financial support, where possible. One patient described how he negotiated his fees with Al-Ibrahim Trust Eye Hospital in Karachi:

“*Only last day [yesterday] they told … me 6500 rupees [approximately US$ 40] will be charged*. *But I pleaded to them that I am a poor man*. *Then they gave me a slip*, *it was filled with the reduced fee*, *and I deposited 4000 rupees [US$ 25]*.*”*

***[Male patient, 65 years, Karachi]***

#### Busy hospitals and long waiting times

Patients described that visiting hospitals was time consuming. Long queues and waiting times were reported to be significant barriers to follow up visits and uptake of referrals. The two public hospitals in particular, were reported to be very busy, with long queues to register and then consult a doctor. For most patients, it took the entire day to visit the hospital. Those who were working or had household duties could not take a full day off. In addition, waiting long hours in a queue was painful for patients with advanced diabetes. Many participants said they were dreading trips to the public sector tertiary facilities and visited them only when the situation was ‘*getting out of control*’. Patients, who needed only simple routine monitoring said that they preferred visiting community GPs, who were available in the evenings and could check blood pressure and sugar level at a small fee.

Patients were particularly reluctant to visit government facilities because they were overstretched. Doctors and other medical staff in these facilities reported to be short of time. This is how one doctor from Lahore described his busy schedule:

“*… we have cases of orbit*, *… tuberculosis*, *patients with glaucoma*, *squint … we see kids*, *adults*, *elderly …*. *If today I start seeing diabetes patients*, *I will have lots and lots of patients with diabetes alone*. *Every day we are doing 12–13 VR [vitreo-retinal] surgeries*, *every day …*. *Here we see a lot of patients with a range of diseases*”.

***[Doctor, Mayo Hospital, Lahore]***

Some clinical procedures, such as dilation of the pupil were also time consuming. To address this challenge and speed up the examination process, the project bought more advanced eye screening technology, such as a non-mydriatric camera, which does not require the dilation of the pupil.

Some respondents from the public hospitals also reported that the patients known to the staff managing the patients’ flow were allowed to bypass the queues, which was very frustrating for those waiting. In some instances, when the hospitals were too full and the working day was over, patients were asked to leave without seeing a doctor and told to come back the next day. Many patients would not come back.

“*But when we have to spend 6 to 7 hours waiting and don’t get treatment*, *and told to come back the next day then it is useless*”.

***[Male patient, 62 years, Lahore]***

#### Difficulties with travel and transportation

Availability of public transportation was reported to be a major factor in the uptake of referrals. The DM/DR unit in Karachi was reported to be difficult to reach via a direct transportation route. Many patients had to change buses two to three times to get to the facility. The trip was particularly challenging for old people and those with advanced diabetes. The costs of private taxis was reported to be too high, and the majority of patients could not afford them. In Lahore and Rawalpindi, the project facilities are located in the middle of the cities. However, busy roads and the lack of public transport was also reported to be a barrier for some patients in these districts.

Female patients were reported to have additional barriers with travelling, as they were required to get permission from their husbands to visit the hospital and also needed someone to accompany them. This is how one female patient in Karachi explained her reasons for not taking up referrals:

“*In our community*, *females cannot move alone*. *I do not want to come with anyone other than my son or daughter in law*. *This was the reason for the delay*”

***[Female patient, 70 years, Karachi]***

#### Lack of awareness about eye related complications

Most patients interviewed in this study were aware of the effects of diabetes on their daily routine and wellbeing as well as complications of diabetes affecting feet, heart or kidneys. They were less aware, however, of the impact of diabetes on eyes and vision. Even those who were reasonably well educated, did not seem to be aware of eye related complications of diabetes. Some of the referred patients said that they had been told about the effect of diabetes on vision only by LHWs.

“*I did not know*. *It is the lady health worker who told me that the problem I had in my eyes was because of sugar*, *my sugar was not getting under controlled*. *So*, *she asked me to come here*. *They checked my eyes yesterday and have suggested laser for both eyes*”.

***[Female patient, 58 years, Karachi]***

## Discussion

This study explored the experiences of patients and health care providers participating in a project, which aimed to improve access to DM/DR care in Pakistan. The project included a broad range of activities, including awareness raising, counselling, referrals from community and primary care providers, strengthening infrastructure and improving integration of services. The findings are broadly consistent with other literature on the uptake of referrals and adherence to treatment in LMICs [10–13]. Some common barriers, such as overcrowded facilities, long waiting times, user fees and the lack of transport, are well known and have been reported in many similar contexts [14–17]. However, this study provides useful insights into how these challenges manifest in DM/DR care and where the pressure points are likely to arise creating bottlenecks in access to healthcare.

Our findings on the referral pathways show that at present, there are a number of ways through which patients with diabetes access treatment services in Pakistan. The majority of patients appear to be self-referred, although it was also clear from the study that seeking care is not an immediate priority for many patients and delays in consulting with healthcare providers often occur.

One of the innovative approaches tried in this project was mobilisation of communities and raising awareness of DM/DR by lady health workers, who are uniquely positioned to link remote rural communities to specialist healthcare services. Our findings however suggest that despite the initial good intentions, LHW engagement created the first pressure point along the continuum of diabetic care. Among primary care providers involved in the project, LHWs appear to be the least effective in securing an uptake of referrals. There are several reasons for this. First, neither the LHWs themselves, nor their supervisors at the local health departments saw eye care as a health priority. LHWs were not required to report on DM/DR or any other eye conditions as part of their training and did not view eye health services as their key responsibility, a finding reported in a number of earlier studies [18, 19]. Second, expanding their scope of work and mandating them to mobilise patients for eye care not only increased their workload, it also pushed them beyond their traditional target population group, which typically consists of women and children [20]. In this project, LHWs could not effectively engage with male members of the household to mobilise them for DM/DR care. Also, we did not find any evidence to suggest that the LHWs’ training and engagement mitigated traditional barriers to healthcare experienced by women in Pakistan, such as permissions and companions to travel [21–24]. Consequently, LHWs were not particularly successful in bringing either male or female patients to the DM/DR care. In addition, even after the training, LHWs were not perceived as very knowledgeable about the complex diseases related to the eye or about the nature of services to be received in tertiary hospitals, which highlights the weaknesses of the training provided.

Given that LHWs are an important and integral part of community healthcare in Pakistan [25, 26], there is great potential in their role in diabetes care—including education of patients, referrals and adherence to treatment and follow up. However, to make their role more effective, more system level changes are required. Diabetes care, including eye care, should be part of their scope of work. They should be adequately trained not only on DM and DR but also on the way the tertiary facilities work. It is important that community health programmes are designed to engage with male household members and community leaders as well as help identifying women, who may experience particular difficulties in accessing health facilities. However, their impact on improving access to care for DM/DR patients will only be felt if these additional activities are balanced with their existing responsibilities and current workload.

Similar to earlier studies [27, 28], this research showed that patients’ decisions on the uptake of DM/DR referrals and treatments, are influenced by a range of socio-economic and health system factors, which create a second pressure point in improving DM/DR care. Poverty and financial constraints have been shown to be an important barrier to seeking care in many settings [29, 30]. Although all three facilities participating in this project had provisions for poorer patients through user fee exemptions or subsidised fees, direct costs of medicines and indirect costs of transport or time off work, were challenging for many patients and prevented them from the referral uptake. Existing literature from other settings highlights a variety of strategies people adopt when delaying seeking care [31, 32]. In this study, participants reported using local medical stores, home treatments and herbal remedies as their first points of care. It may be useful to explore how these existing local opportunities can be better used in health education and behaviour change communication [33].

This project involved both public and private sector providers and described the differences in the way they deliver care. A third pressure point in delivering integrated DR/DM care resides in how these modes of delivery impact patients’ perceptions and experiences of services and subsequently referral and treatment uptake. Unsurprisingly, the private non-for-profit hospital with less overcrowded facilities, less overworked staff and more conveniently arranged services provided on one site, was perceived to be of higher quality and patients seemed to be happier about the care they received there. On the other hand, the two government hospitals with large numbers of patients, long queues, inconveniently located services and uncomfortable premises were a deterrent of hospital visits, even though most patients appreciated the free care and the professionalism of staff in these facilities. Study findings clearly show that smaller and more convenient health facilities, located closer to patient homes and open at convenient times, are likely to increase the uptake of referrals and follow ups. In this study for example, some patients preferred to visit their GPs for blood pressure and glucose level measurements, even though they had to pay for these services. These findings are in line with other research in South Asia and elsewhere, which shows that many patients associate good quality care with cleanliness of premises, convenient location and opening times and positive attitudes of staff [34, 35]. Opportunities for developing screening and treatment services for DM/DR in smaller, more conveniently located facilities need to be explored. The use of mobile facilities and technologies should also be explored to facilitate this alternative type of care.

Some of the results in our study point to the potential of three components of the DM/DR integration that should be taken into account in designing DM/DR interventions. Firstly, counselling for DM/DR patients was shown to be important and should not be neglected. It is important that the project facilities find ways to sustain these services beyond the life of the project. Such counselling sessions should also consider patients’ personal circumstances and social context [36]. As mentioned, women were found to be highly dependent on their husbands not only for financial resources, but also for permission to travel and consult a doctor. Counselling sessions should therefore not only target the patients but also the key decision-makers of their households.

Secondly, an important initiative of this project was a patient tracking database, which helped to monitor patients’ referrals and follow ups in a more systematic way and supported the automated reminders of the appointments sent to patients’ mobile phones. The system was thought to be effective from both patient and provider perspectives and it is important that the project hospitals find a way to maintain these systems beyond the life of the project.

Thirdly, the cross-departmental linkages and collaboration established by the project in the three participating hospitals were thought to be beneficial for both patients and healthcare staff. The linkages seemed to ensure continuity of care and improve institutional governance and coordination. Literature suggests that the institutional changes of this nature become sustainable when they are fully integrated within the organisational culture, structures and processes [37]. An assessment of cross-departmental linkages and referral systems after the project, will be beneficial to better understand to which extent the three project facilities managed to sustain the benefits they gained.

Our study had a number of limitations. While we collected qualitative data on people’s experience of uptake of referral and treatment, we did not measure the uptake of referrals or treatment in these three hospitals quantitatively. A detailed analysis of the project database will be necessary to make more definitive conclusions on the differences reported during the interviews. Also, we did not have data on patients’ characteristics in terms of their education, socio-economic status or stage of the disease and therefore cannot say whether these individual factors affected patients’ referral pathways or the facilities they accessed. Such analysis will be necessary in the future to make conclusions on the drivers of patient health seeking behaviour and demand for diabetic care.

## Conclusion

The project made significant efforts to improve the response to the growing threat of diabetes in Pakistan. It sought to build health system capacities to identify, refer and treat patients at risk of DR at the primary, secondary and tertiary levels. It provided insights into strengths and weaknesses of different patient referral pathways and care delivery settings. Findings point to the need for services which are patient-centred, i.e., responsive to patients’ needs particularly in term of access, affordability and quality of services provided. Mobile services and innovative technologies should be considered as part of alternative service delivery options. There is also a need for strengthening links with community providers, such as LHWs, which will involve several systemic changes to make their services more effective.

## Supporting information

S1 Table Open-ended questions used during in-depth interviews and focus group discussions with the different categories of study participants. (DOCX)
