# Supplementary material for: Diabetic retinopathy service delivery and integration into the health system in Pakistan—Findings from a multicentre qualitative study
Source: PLoS One. 2021 Dec 15;16(12):e0260936. doi: 10.1371/journal.pone.0260936 (PMC8673653; doi:10.1371/journal.pone.0260936)
Supplement: S1 Table — (DOCX) [file pone.0260936.s001.docx]

| **S 1 Table. Open-ended questions used during in-depth interviews and focus group discussions with the different categories of study participants** | |
| --- | --- |
| **Specific objective** | **Questions** |
| 1. Understanding factors effecting adherence to the referrals in the community | **For community members, referred patients from the community**   - How do you get to know about eye health services in nearby areas? (sources of information and referrals and see if the LHW is mentioned/discussed unprompted) - What role do the LHWs play in the community, what are their key jobs, duties? What is their job in the eye health care/services in your community? - Have you or any one of your relatives/somebody you know been screened, identified/diagnosed with any eye aliment by the LHW? Who? Which disease?. - What was LHW’s advice after diagnosis? What was the process (see if referral slip is mentioned?) - Did you or your relative/somebody you know adhere to the LHWs advice? - if yes WHY (*Moderator/interviewer to probe for motivational factors for adherence to the advice of the LHW)* - If not WHY NOT? *(Moderator/interviewer to explore the barriers for non-adherence to the advice of the LHW)* - How was the experience, in term of access, services, and additional supplies for surgery, post-surgical care, etc*.? For those who adhere to the LHWs advice and visited eye care facility.* |
|  |  |
| 2. How the patient treatment pathways work in practice in each of the three sites and describe how it differs from original design. | **For eye health care staff**   - What is your role in the eye health care project? - How long have you been serving in this department? - What are the standard procedures or the steps to follow in the eye health care in this project (i.e. from community level screening to the high-end service delivery) - Do you think that all the staff members follow the same procedures (from primary staff to technical staff, (optometrist,) and professional staff (like doctors, ophthalmologist, project manager/coordinator and national coordinator)? - How are the current procedures and steps (pathways) different (or similar) to the previous procedures in place prior to the start of this DM/DR project? - What are the advantages and disadvantages of the current procedures and practices compared to the previous practice? |
| 3. Explore integration within services and understand patient and staff experiences of different models and levels of integration. | **For staff and patients**   - How do you feel about one window services under the same umbrella? - How helpful have been the linkages and integration of services to the patients (like coordination between OPD, Gynaecology, General medicines, Nephrology, Diabetes, foot care and eye care) in getting maximum benefit? What are the advantages? - As a staff of hospital (mention department) do you find any challenge being part of this integrated system? - As a patient what are the advantages of linkages between the departments and integrated system? - Do you find any challenges in getting help from DM/DR project? |
|  |  |
| 4. In relevant sites (Lahore Karachi), explore the development and establishment of multi-disciplinary working and understand patient and staff experiences of services delivered this way. | **For patients from the community**   - Have you ever visited the eye health care service delivery unit in the hospital (name of the hospital in each city)? - How often do you visit the service delivery (eye health care) unit? - How do you see the development and establishing one window unit? What is your feedback? - How do you find the range and quality of the services provided under one umbrella unit (DM/DR), and the presence of optometrist, Counsellor, Medical officer, ophthalmologist, surgeon podiatrist (foot care unit), and other staff? - In your opinion what are the best features of the services? - Do you feel there are any shortfalls or gaps in the serves or any areas needing improvement in the DM/DR and other eye healthcare services, as per your experience (as a services seeker)? - What recommendations you suggest to improve the eye healthcare services delivery?   *(Moderator to probe to have deeper understanding of the issues and recommendations for improvement)* |
| 5. Explore how LHWs perceive their role with respect to DM, DR and primary eye care generally, and how well equipped they are to fulfil it. | **For lady health workers (LHWs)**   - What are your roles and responsibilities towards community as LHW? - How long you have been working as LHW and at present duty station? - Did you get any training during the job to better perform your duties/designated role and responsibilities? - Do you know about the eye health care DM/DR project? - What is your role in the DM/DR service delivery? - How long you have been associated to the project? - Did you ever get any training to perform your duties/ services in eye health care? (**ASK** only if not reported earlier in response to trainings probe) - What are the skills you have learnt during the training on eye health care for better service delivery? - Did you get any other support from the DM/DR project in delivering your job (like equipment, technical kit, any other)? - Who trained you? - How do you rate the eye care training? (*Moderator may ask to rate on 1-10 scale*), what are the best features of the training, any short comings in the training session? - Did you get any reference material to remind you about the standard procedures or steps to follow in your eye care job? - How could the training you received be further improved to help you deliver eye health care services? |
|  |  |
| 6. Explore facilitating and constraining factors associated with patient’s compliance to treatment. | **For LHWs**   - Based on your community work experience as LHW, what do you think are the key factors that facilitate the adherence of the eye patients to referrals and treatment   (*probe for age, gender, affordability, type of eye disease, level of education, distance from the service centre, attitude, any other factor*)  What are common factors for majority of the patients as per your experience     - What do you think are the factors that restrict the patient’s adherence to the referral for treatment (*probe for age, gender, affordability, type of eye disease, level of education, distance from the service centre, attitude, and any other factor*)? |
|  |  |
|  | The interviewer may ask any other question(s) that might emerge during the interview and is/are relevant to the research objectives? |
|  | Any other questions/ comments from the participant |
